# A Different Approach of Dizziness in Older Patients: Away from the Diagnostic Dance between Patient and Physician

**DOI:** 10.3389/fmed.2014.00050

**Published:** 2014-12-01

**Authors:** Otto R. Maarsingh, Hanneke Stam, Henriëtte E. van der Horst

**Affiliations:** ^1^Department of General Practice and Elderly Care Medicine, VU University Medical Center, Amsterdam, Netherlands; ^2^EMGO Institute for Health and Care Research, VU University Medical Center, Amsterdam, Netherlands

**Keywords:** dizziness, older people, prognosis, diagnosis, general practice

## Abstract

Although the etiology of dizziness in older patients differs significantly from that of younger patients, most guidelines on dizziness advocate the same diagnosis-oriented approach for all patients regardless of their age. However, this diagnosis-oriented approach may be insufficient for older patients presenting with dizziness in general practice, because (1) general practitioners are often not able to identify an underlying cause of dizziness, (2) general practitioners regularly identify causes of dizziness that cannot be treated, and (3) general practitioners may identify causes of dizziness for which treatment is available but not desirable. In this article, the authors present a simultaneous diagnosis- and prognosis-oriented approach for older dizzy patients. This approach may enable general practitioners to improve their care for a voluminous group of impaired older patients, even if a diagnosis is not available (yet).

Dizziness – like falling and incontinence – is one of the geriatric giants. Thirty percent of people above 65 years of age experience some form of dizziness, increasing to 50% in persons above 85 years (1). Nine percent of all persons above 65 years visit their general practitioner (GP) at least once a year because of dizziness (2). The complaint dizziness can lead to severe limitations in daily functioning (3). Dizziness is also a major risk factor for falling (4), thus leading to further limitation of activities, fractures, and high healthcare costs (5).

Although the etiology of dizziness in older patients differs significantly from that of younger patients, most guidelines on dizziness advocate the same diagnosis-oriented approach for all patients regardless of their age. However, this diagnosis-oriented approach may be insufficient for older patients presenting with dizziness. Firstly, GPs are often not able to identify an underlying cause of dizziness [40–80% of older dizzy patients in general practice; (2, 6)]. Secondly, GPs regularly identify causes of dizziness that cannot be treated (e.g., polyneuropathy, 19% of older dizzy patients in general practice) or hardly treated [e.g., orthostatic hypotension, 24% of older dizzy patients; (7)]. Finally, GPs may identify causes of dizziness for which treatment is available but not desirable [e.g., Epley maneuver for benign paroxysmal positional vertigo in patients with severe cervical arthrosis; (8)].

When applied to older patients with *chronic* dizziness [70% of older dizzy patients in primary care have a dizziness onset of ≥6 months ago; (7)], the current diagnosis-oriented approach is not only insufficient but may also have negative side-effects. When a GP is not able to identify an underlying cause of dizziness, patient and GP may persist in a diagnostic dance, leading to extensive testing and unnecessary referral (9, 10). An absent diagnosis and – consequently – clues for therapeutic interventions may also lead to inappropriate drug prescribing. Data from the Second Dutch National Survey of General Practice (DNSGP-2) show that GPs prescribed ineffective drugs to 10% of dizzy older patients during the first consultation (11). Eventually, a GP may indulge in therapeutic nihilism: “dizziness comes with age” or “you will have to live with this.”

Previously, other researchers emphasized the limitations of the current diagnosis-oriented approach of dizziness. In 1999, Sloane and Dallara already expressed the need for strategies that more effectively reduce symptoms and dizziness-related disability (12). In 2000, Tinetti and others suggested that considering dizziness, a geriatric syndrome might lay the groundwork for such an impairment reduction strategy (3). Geriatric syndromes are multifactorial health conditions that occur when the accumulated effect of impairments in multiple systems renders a person vulnerable to situational challenges. More recently, other researchers supported this concept of dizziness as a geriatric syndrome (13–16).

When focusing on underlying causes of dizziness, it is easy to neglect individual prognostic differences. Dros and others found that 34% of older dizzy patients in general practice will experience persistent dizziness-related impairment after 6 months (17). They found dizziness-related impairment at baseline [as measured with the Dizziness Handicap Inventory, DHI; (18)] to be the strongest predictor of persistent dizziness-related impairment. Additionally, they identified seven predictors of persistent dizziness-related impairment, namely (1) dizziness onset of more than 6 months ago, (2) dizziness provoked by standing still, (3) associated trouble with walking, (4) polypharmacy, (5) absence of diabetes mellitus (explanation: diabetes may be a marker for attentive medical care), (6) presence of anxiety or depression, and (7) impaired functional mobility (17). The research group also constructed a seven-item sum score to calculate the probability of persistent dizziness-related impairment (17). An example, when applying the score calculation in a patient X with chronic dizziness provoked by standing still and associated with walking, using six drugs and having a comorbid depression and impaired functional mobility, the probability of persistent dizziness-related impairment in patient X changes from 34 to 85%. Even if patient X’s GP is not able to identify an underlying cause of dizziness (yet), patient X has three predictors of persistent impairment that are amenable to treatment, namely (1) polypharmacy (treatment: medication reduction), (2) depression (psychotherapy and/or antidepressants), and (3) impaired functional mobility (physiotherapy).

For the clinical approach of *chronic* dizziness in older patients in primary care, we would like to suggest to replace the current “serial connection” between diagnosis and prognosis by a “parallel connection,” i.e., the diagnosis-oriented phase and prognosis-oriented phase start at the same time (see Figure 1). Currently, GPs are used to focus on prognosis *after* diagnosing a specific disease, for example, investigating the presence of albuminuria – as a marker for kidney disease – after diagnosing diabetes mellitus. In other words, the prognosis-oriented phase (“is this patient at risk of an unfavorable outcome and, if so, how to improve this outcome?”) follows the diagnosis-oriented phase (“what is the underlying cause of the presented complaint?”). We believe that the suggested “parallel connection” of diagnosis and prognosis is crucial for older dizzy patients in primary care, because many older dizzy patients remain undiagnosed but have clues for impairment reduction (3, 13, 14).

**Figure 1:**
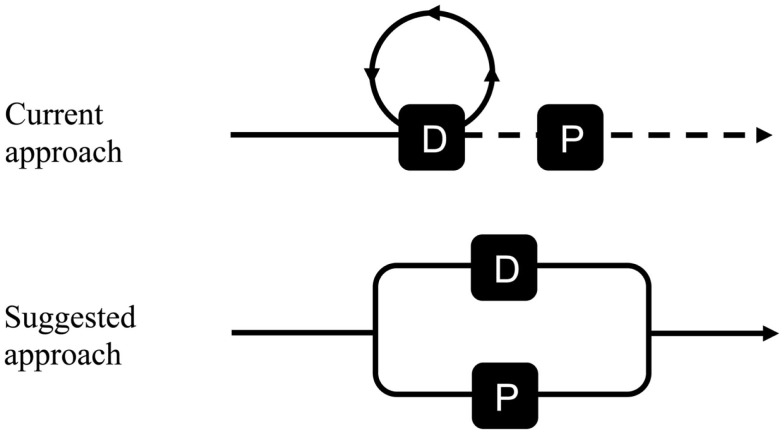
**Schematic representation of current and suggested approach of chronic dizziness in older patients in general practice (D, diagnosis-oriented phase; P, prognosis-oriented phase)**.

For daily clinical practice, the suggested additional prognosis-oriented approach could imply that a GP tries (1) to estimate if an older dizzy patient is at risk of persistent impairment [by using the DHI and the seven-item sum score; (17, 18)] and, if so, (2) to identify modifiable predictors of an unfavorable course of dizziness. Examples of previously identified modifiable predictors are impaired functional mobility [treatment: physical exercise or physiotherapy; (17)], comorbid anxiety [psychotherapy and/or anxiolytics; (17, 19)], comorbid depression [psychotherapy and/or antidepressants; (17)], dizziness due to psychiatric causes [psychotherapy and/or psychotropic drugs; (20)], polypharmacy [medication reduction; (17)], and the presence of avoidance [cognitive behavioral therapy; (21)].

Finally, we do not want to advocate diagnostic nihilism. The suggested approach should not be at the expense of a thorough diagnostic evaluation. Benign paroxysmal positional vertigo, for example, is a cause of dizziness in older patients that can be easily diagnosed [history taking and Hallpike maneuver; (22)] and effectively treated [Epley maneuver; (8, 23)]. The clinical approach of dizziness in older patients requires personalized care, though, in which it is necessary to find a delicate balance between excessive testing and diagnostic nihilism.

## Conclusion

The current diagnosis-oriented approach does not suit older patients presenting with dizziness. A simultaneous diagnosis- and prognosis-oriented approach for older dizzy patients may enable GPs to improve their care for a voluminous group of impaired older patients, even if a diagnosis is not available (yet). Hopefully, such an approach will not only improve the patient’s quality of life, but also reduce inappropriate drug prescribing and unnecessary referral.

## Conflict of Interest Statement

The authors declare that the research was conducted in the absence of any commercial or financial relationships that could be construed as a potential conflict of interest.
